# The homeodomain transcription factor Orthopedia is involved in development of the *Drosophila* hindgut

**DOI:** 10.1186/s41065-020-00160-y

**Published:** 2020-11-19

**Authors:** Kirsten Hildebrandt, Nicole Bach, Dieter Kolb, Uwe Walldorf

**Affiliations:** Developmental Biology, Saarland University, Building 61, 66421 Homburg/Saar, Saarland Germany

**Keywords:** *Drosophila* hindgut, Orthopedia, Transcription factor, Homeobox, *otp* mutants

## Abstract

**Background:**

The *Drosophila* hindgut is commonly used model for studying various aspects of organogenesis like primordium establishment, further specification, patterning, and morphogenesis. During embryonic development of *Drosophila*, many transcriptional activators are involved in the formation of the hindgut. The transcription factor Orthopedia (Otp), a member of the 57B homeobox gene cluster, is expressed in the hindgut and nervous system of developing *Drosophila* embryos, but due to the lack of mutants no functional analysis has been conducted yet.

**Results:**

We show that two different *otp* transcripts, a hindgut-specific and a nervous system-specific form, are present in the *Drosophila* embryo. Using an Otp antibody, a detailed expression analysis during hindgut development was carried out. Otp was not only expressed in the embryonic hindgut, but also in the larval and adult hindgut. To analyse the function of *otp*, we generated the mutant *otp* allele *otp*^GT^ by ends-out gene targeting. In addition, we isolated two EMS-induced *otp* alleles in a genetic screen for mutants of the 57B region. All three *otp* alleles showed embryonic lethality with a severe hindgut phenotype. Anal pads were reduced and the large intestine was completely missing. This phenotype is due to apoptosis in the hindgut primordium and the developing hindgut.

**Conclusion:**

Our data suggest that Otp is another important factor for hindgut development of *Drosophila*. As a downstream factor of *byn* Otp is most likely present only in differentiated hindgut cells during all stages of development rather than in stem cells.

## Background

The *Drosophila* embryonic hindgut is a single-layered ectodermally derived epithelium surrounded by visceral musculature [1–3]. It arises from a group of cells at the posterior part of the blastoderm stage embryo referred to as the hindgut primordium [1]. The hindgut primordium is a ring of about 200 blastoderm cells that is internalised during gastrulation to form a short, wide sac. In a relatively short time this epithelium sac is transformed into a long tube containing approximately 700 cells [1, 4, 5]. The growth of the hindgut starting at stage 12 is not due to cell divisions, but a twofold endoreplication [6] that leads to an increase in cell size, and as a consequence total length of the hindgut [7, 8]. During this process, the developing hindgut becomes subdivided along the anterior posterior (AP) axis and the dorsoventral (DV) axis. Along the AP axis, the hindgut forms three morphologically distinct regions: the small intestine, large intestine, and rectum [5, 8, 9]. The small intestine is the most anterior part of the hindgut and is connected to the posterior midgut, whereas the large intestine is the central part of the hindgut and forms three distinct regions along the DV axis. The dorsal and ventral regions constitute the outer and inner portions of the hindgut loop, respectively. Two rows of boundary cells are organised between these two regions and as two rings at the anterior and posterior borders of the large intestine [8, 10, 11]. The most posterior-most portion of the hindgut is the rectum, which connects to the anal pads.

Several genes are required to establish the hindgut primordium and to pattern the hindgut along the AP axis. At the blastoderm stage a group of posterior cells, called the proctodeal primordium (Campos-Ortega and Hartenstein, 1997) [1], will later on give rise to the hindgut. In these cells the transcription factor Tailless (Tll) [12] is expressed and subsequently activates other transcription factors like Brachyenteron (Byn) [13], Fork head (Fkh) [14] and Bowel (Bowl) [15] as well as the signalling protein Wingless (Wg) [16], which are all necessary for hindgut development. The transcription factor Caudal (Cad) [17] is also expressed in the proctodeal ring, but independently of Tailless. Tll [18] and Wg [9, 19, 20] are necessary to establish the primordium, whereas Cad is necessary for the internalisation of the hindgut primordium later on [21]. Proper gene expression in and maintenance of the large intestine requires *byn*, *Dichaete* (*D*), *raw*, *lines* (*lin*) and *mummy* (*mmy*), while *bowl* and *drumstick* (*drm*) are required for gene expression in and maintenance of the small intestine [5, 19, 22–26].

The *Drosophila* T-box gene *brachyenteron* (*byn*) is expressed in the ring of cells that internalise to form the embryonic hindgut and expression is maintained in the hindgut throughout embryogenesis [13]. In *byn* mutants the hindgut is shortened due to apoptosis and the large intestine is missing [11, 22]. The *Drosophila* homeobox gene *orthopedia* (*otp*) is also expressed in the hindgut, anal pads and along the central nervous system [27]. It is located in 57B region of the second chromosome in close vicinity to the other homeobox genes *Drosophila retinal homeobox* (*Drx*) [28, 29] and *homeobrain* (*hbn*) [30]. In the hindgut, *otp* is directly activated by *byn* in a dose-dependent manner via multiple binding sites present in a regulatory element of *otp* [31].

Otp is highly conserved through evolution and has been identified in most multicellular organisms. Among these are several invertebrates such as sea urchins [27], the mollusc *Patella vulgata* [32], the annelid *Platynereis dumerilii* [33] and several vertebrates such as zebrafish, that have two genes namely *otp1* and *otp2* [34], chicken [27, 35], mouse [27] and human [36]. Otp genes of vertebrates have a major function in the development of the hypothalamic neuroendocrine system (see [37] for review).

The function of *otp* during *Drosophila* development has been unknown so far as no mutants have been described. In the present study, we show that *otp* is required for proper hindgut development in *Drosophila*. We generated one *otp* allele by ends-out gene targeting and isolated two additional *otp* alleles in an EMS-mutagenesis screen. All three *otp* alleles are characterised by a dramatically reduced hindgut lacking the complete large intestine. This reduction in hindgut length is due to apoptosis in the hindgut primordium and the developing hindgut.

## Results

### Hindgut and nervous system specific transcripts of the *otp* gene

**Fig. 1:**
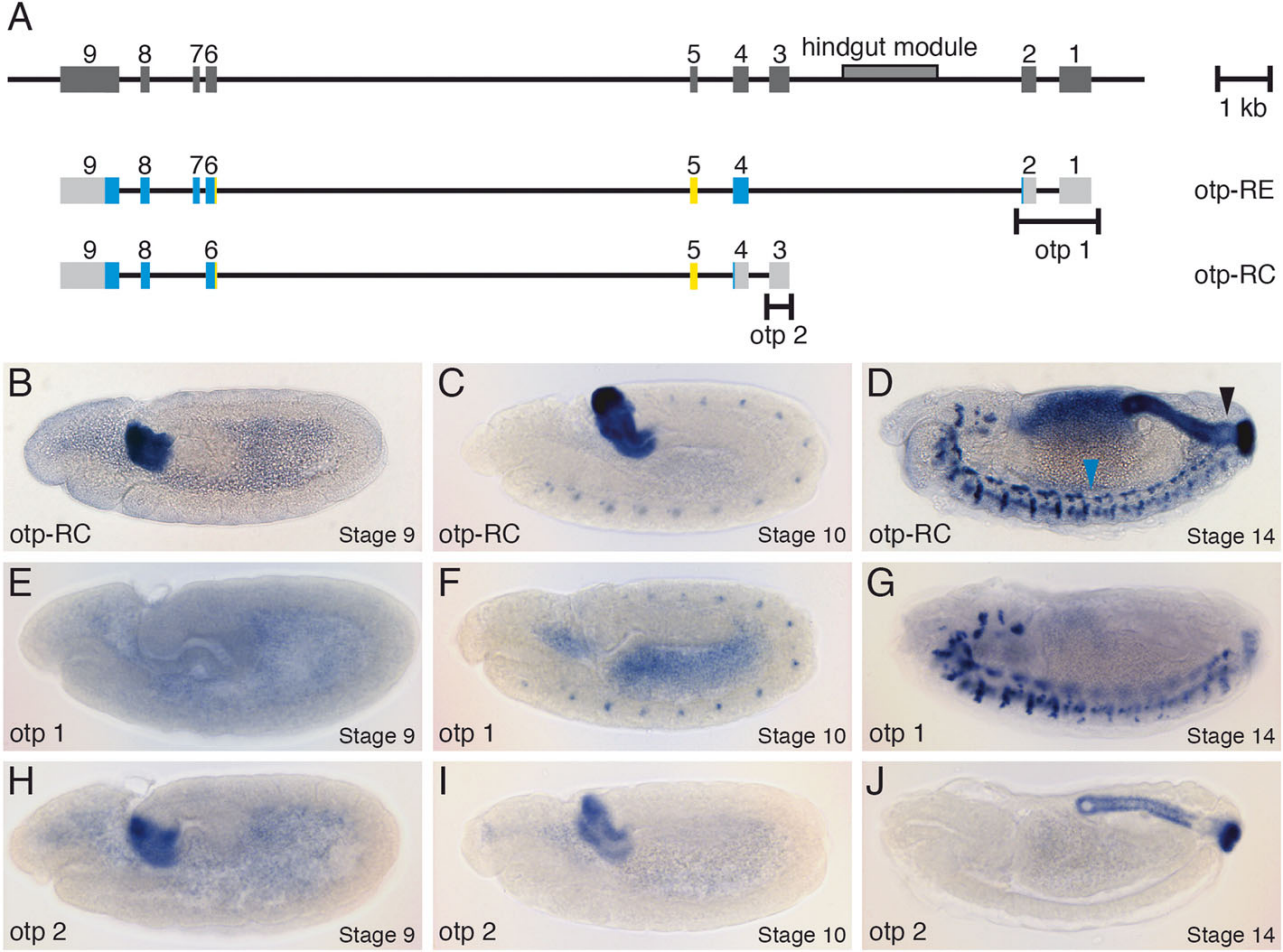
Hindgut and nervous system specific transcripts of the *orthopedia* (*otp*) gene. **a** Genomic organisation of the *otp* gene with exons indicated as gray boxes and the regulatory region for the *otp* hindgut expression with the Byn binding sites (hindgut module) [31]. Below the two different transcripts (*otp*-RE and *otp*-RC) are shown (gray, untranslated region; blue, translated region; yellow, homeobox). The specific genomic probes (otp 1 and otp 2) used to detect *otp*-RE and *otp*-RC by in situ hybridisation are indicated. **b**-**j** Embryonic expression pattern of the different *otp* transcripts. The stages shown (9, 10, and 14) were determined according to Campos-Ortega and Hartenstein (1997) [1], all views are lateral views, anterior is to the left. **b**-**d** Whole-mount in situ hybridisation performed with an *otp* cDNA probe against exons 3–9 detecting all transcript forms (otp-RC). The boundary between stronger anterior expression and weaker posterior expression in the nervous system is indicated by a blue arrowhead, the weaker expression in the rectum compared with the rest of the hindgut by a black arrowhead in **d**. **e**-**g** In situ hybridisation performed with a probe against exon 1 and 2 of the *otp* gene (otp 1). **h**-**j** In situ hybridisation performed with a probe against exon 3 of the *otp* gene (otp 2)

The expression of the *otp* gene during embryonic development was first described by Simeone et al. (1994) [27]. The gene is expressed first in the hindgut primordium, then in the hindgut and anal pads as well as in the ventral nerve cord and in the brain of the *Drosophila* embryo. With the help of cDNAs and ESTs which have been analysed over the years nine different transcript forms of *otp* are known today (Flybase FB2020_03, [38]). They basically fall into two different classes, some which have exons 1 and 2 spliced to exon 4, here *otp*-RE is an example, and others which have exon 3 spliced to exon 4, here otp-RC is one representative (Flybase FB2020_03, [38]) (Fig. 1a). A 2.3 kb transcript (referred to as *otp*-RC by Flybase) can first be detected in 3–6 h old embryos. In 9–12 h old embryos an additional 2.9 kb transcript (referred to as *otp*-RE by Flybase) can be detected. The expression of both transcripts remains throughout all embryonic and larval stages.

Because the two *otp* transcripts show different temporal expression we contemplated whether they also differ in their spatial expression. Since the two *otp* transcripts vary in the 5′ part, exons 1 and 2 are specific to the larger 2.9 kb transcript, exon 3 is specific to the smaller 2.3 kb transcript, and each transcript can be detected with a specific probe.

After analysing whole mount in situ hybridisations of embryos with a probe detecting exons 3–9 sequences (probe otp-RC) that are present in both transcripts, the *otp* expression was mostly consistent with that described by Simeone et al. (1994) [27]. The earliest expression was visible in the hindgut primordium, the proctodeum, at stage 9 (Fig. 1b), an additional *otp* expression in the nervous system begins at stage 10 (Fig. 1c) and *otp* is expressed in the hindgut, anal pads, ventral nerve cord and brain at stage 14 (Fig. 1d). Whereas Simeone et al. (1994) [27] detected no expression of *otp* in the rectum, we detected *otp* expression in this small portion at the most posterior part of the hindgut, albeit at a lower level than in the other parts of the hindgut (Fig. 1d, black arrowhead). In the nervous system, there was a clear border with a higher level of *otp* expression in the five most anterior segments of the ventral nerve cord and a lower level of *otp* expression in the posterior segments of the ventral nerve cord (Fig. 1d, blue arrowhead indicating the border).

Using a probe against exons 1 and 2 of the *otp* gene (probe otp 1), no expression was detected in stage 9 embryos (Fig. 1e). At stage 10, *otp* expression was only visible in the nervous system, but not in the hindgut primordium (Fig. 1f). At stage 14, *otp* expression was again only present in the ventral nerve cord and brain (Fig. 1g).

With a probe against exon 3 of the *otp* gene (probe otp 2), the complementary expression pattern can be detected. At stage 9 *otp* expression occurs in the hindgut primordium (Fig. 1h), and at stage 10, *otp* expression continues embryos also in the hindgut primordium, but not in the nervous system (Fig. 1i). At stage 14, *otp* expression can be detected in the hindgut and anal pads, but not in the ventral nerve cord and brain (Fig. 1j).

These results indicate that the smaller 2.3 kb *otp* transcript is specifically expressed in the hindgut primordium, embryonic hindgut and anal pads, whereas the expression of the larger 2.9 kb *otp* transcript is restricted to the embryonic nervous system, where it is expressed in the ventral nerve cord and brain.

### Otp expression during embryonic development

**Fig. 2:**
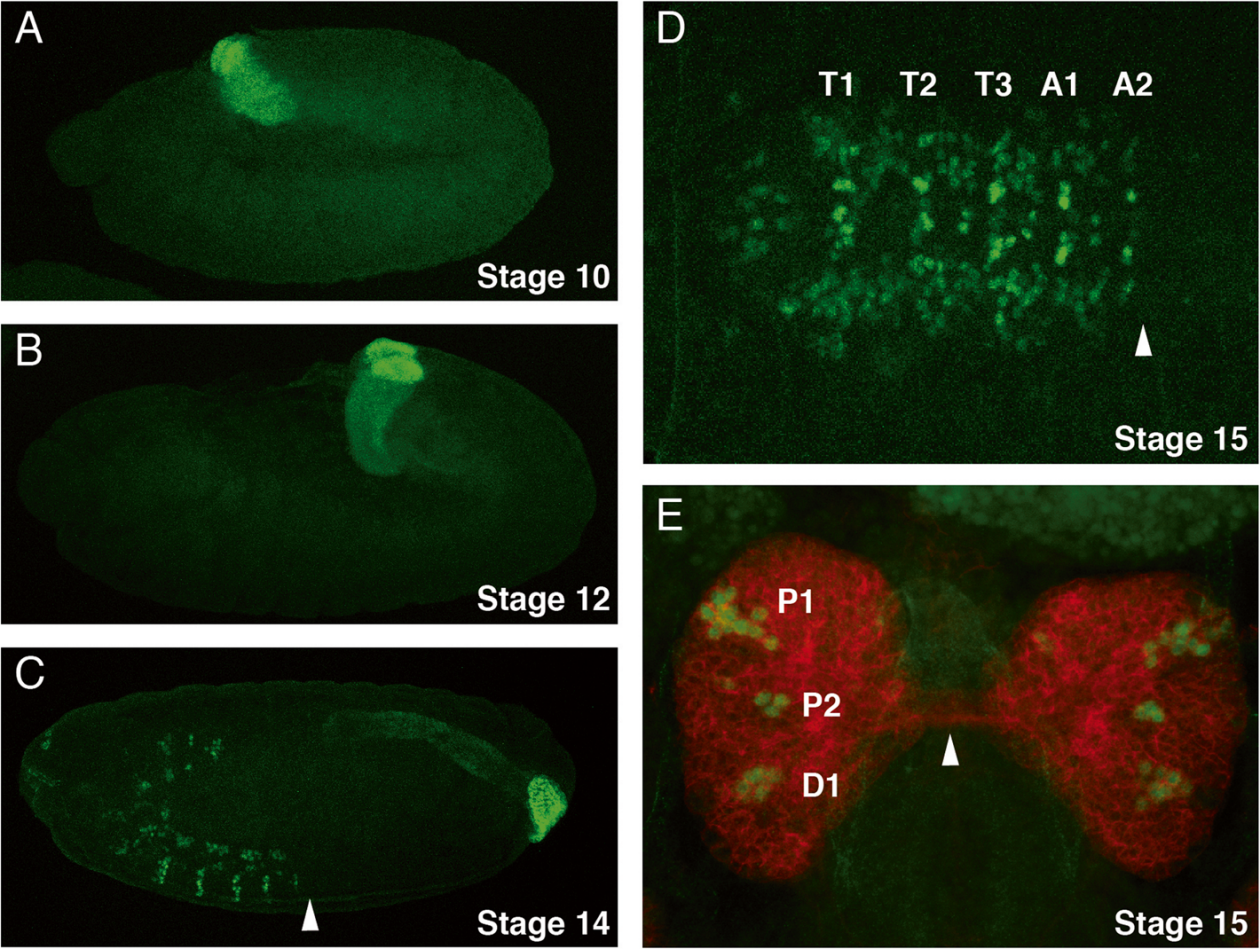
Expression of Otp during embryonic development. Laser confocal images of *Drosophila* embryos labeled with an anti-Otp antibody (green). Stages were determined according to Campos-Ortega and Hartenstein (1997) [1] as indicated in the figure. **a**-**c** Lateral views of embryos, the anterior ends are to the left. In **c** the expression border in the ventral nerve cord is shown by an arrowhead. **d** Ventral view of the embryonic nerve cord, segments T1-A2 are labeled and the border of the nervous system expression is again indicated by an arrowhead. **e** Dorsal view of an embryonic brain stained with anti-Otp (green) and anti-Nrt antibodies (red) to visualize the primary neurons, the Otp expression domains are labelled P1 (protocerebrum), P2 (protocerebrum) and D1 (deutocerebrum). The supraesoghageal commissure is indicated by an arrowhead

In order to analyse the Otp protein expression at a higher resolution, we produced an antibody against the Otp-C-terminus in guinea pigs. As seen in the case of the otp mRNA, protein expression started in the proctodeum at stage 10, which was detectable in nuclei as expected (Fig. 2a). During germband retraction at stage 12 the developing hindgut already had the shape of a small tube and the anal pads were visible (Fig. 2b). When the germband retraction was finished at stage 14 expression was seen in the hindgut and the anal pads, but in addition also in the brain and ventral nerve cord (Fig. 2c). One major difference between the protein expression and mRNA expression was detectable in the ventral nerve cord, where the protein is only expressed up to segment A2 (Fig. 2c, white arrowhead) and not in the more posteriorly located segments. This was even better visible in a ventral view of the nerve cord at stage 15 (Fig. 2d, white arrowhead). In the embryonic brain, expression was seen in three discrete expression domains per hemisphere, labeled P1, P2, and D1 from anterior to posterior with respect to the neuraxis (Fig. 2e). Compared to the supraesoghageal commissure seen by the Nrt staining (white arrowhead), P1 and P2 were located in the protocerebrum and D1 in the deutocerebrum. The expression domains consisted of 10 (P1), 3 (P2) and 5 (D1) neurons (Fig. 2e).

### Expression of Otp in the embryonic, larval and adult hindgut

**Fig. 3:**
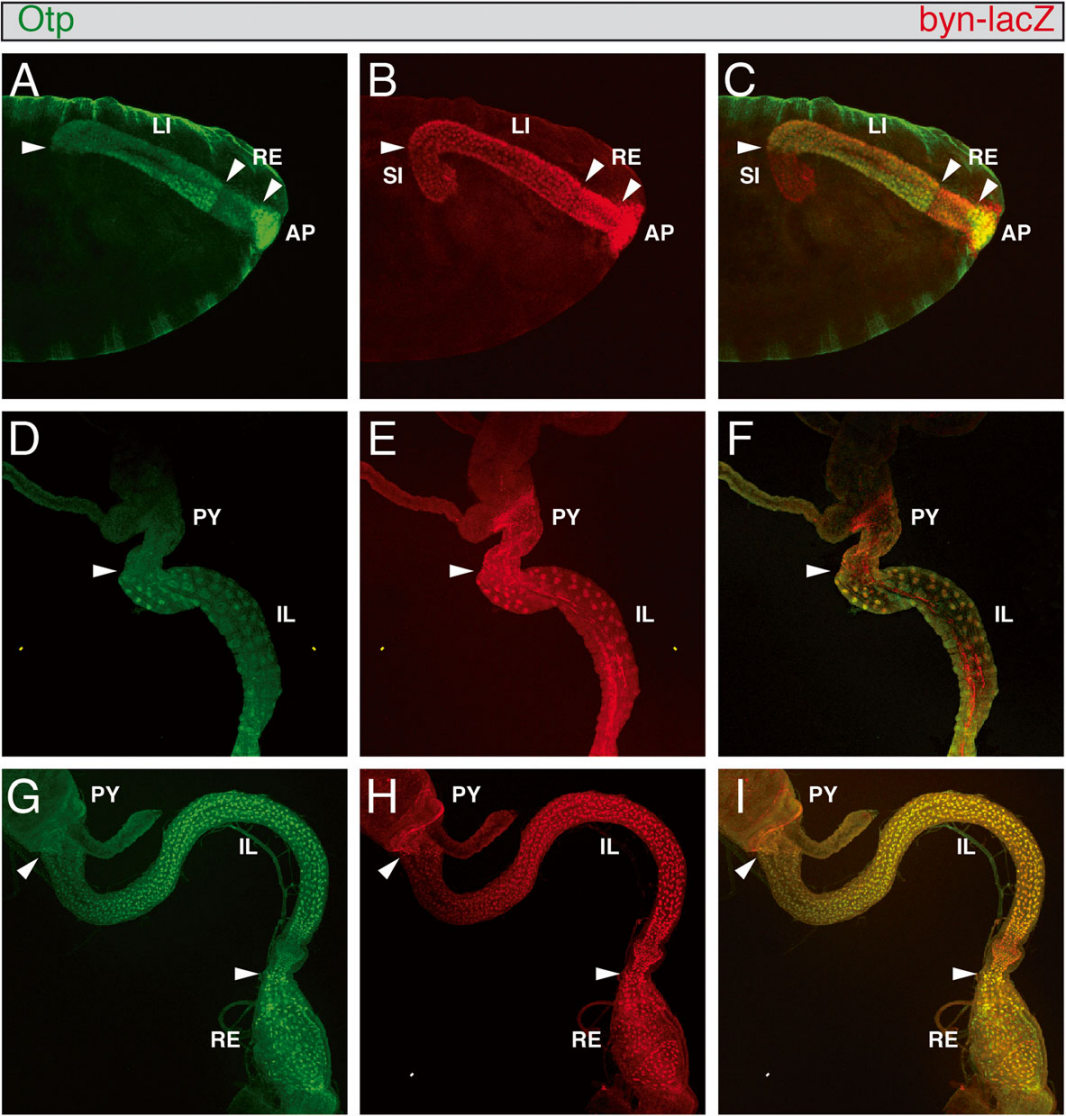
Embryonic, larval and adult hindgut expression pattern of the Otp protein. Anti-Otp antibody staining in green (**a**, **d**, **g**), anti-β-Galactosidase antibody staining of *byn*^apro^ in red (**b**, **e**, **h**) and merging of both stainings (**c**, **f**, **i**). In all views, anterior is to the left, embryos are shown as lateral views. **a**-**c** stage 16 embryo, **d**-**f** larval hindgut, **g**-**i** adult hindgut. The borders between different structures are indicated by white arrowheads. AP, anal pads; HG, hindgut; IL, ileus; LI, large intestine; PY, pylorus; RE, rectum; SI, small intestine

Like *otp* mRNA, Otp protein was expressed in the embryonic hindgut and anal pads at embryonic stage 14 (Fig. 3a). *Byn* is a direct regulator of *otp* and sufficient to induce *otp* expression [31]. The enhancer trap line *byn*^apro^ which mimics Byn protein expression [10] was therefore used to investigate in which parts of the hindgut Otp and Byn are co-localised. Otp and Byn showed co-localisation in large parts of the hindgut primordium (data not shown) and along the complete large intestine (Fig. 3a-c), whereas in the small intestine, only Byn was expressed. Both Otp and Byn are also co-expressed in the rectum, but as for the *otp* mRNA the level of Otp protein in this part of the hindgut was lower than in the large intestine and anal pads (Fig. 3a-c). By contrast, the level of Byn expression did not differ between the different parts of the hindgut. Byn and Otp were also co-localised in the anal pads with an additional outer ring of cells only expressing Byn (Fig. 3a-c). Both the larval and the adult hindgut were subdivided along the anterior-posterior axis into three segments: the pylorus, ileum, and rectum. The most anterior part, the pylorus, which is also called the hindgut proliferation zone (HPZ), is an important region for the formation of the adult hindgut, because the cells of the larval hindgut undergo apoptosis during metamorphosis and are replaced by cells of the HPZ to form the adult hindgut [39]. In our study, in the larval hindgut, Otp and Byn were co-expressed in most parts (Fig. 3d-f). The only exception here was again the most anterior region, the pylorus, where only Byn was expressed (Fig. 3e), but not Otp (Fig. 3d). The co-expression of Otp and Byn was also visible in the adult hindgut (Fig. 3g-i). Here, as in the larval hindgut, Otp was only expressed in the ileum and rectum, whereas Byn was additionally expressed in the pylorus. The expression of both factors suggested a role not only in embryonic and larval hindgut development, but also in maintenance of the adult hindgut as well.

### Generation of *otp* alleles

**Fig. 4:**
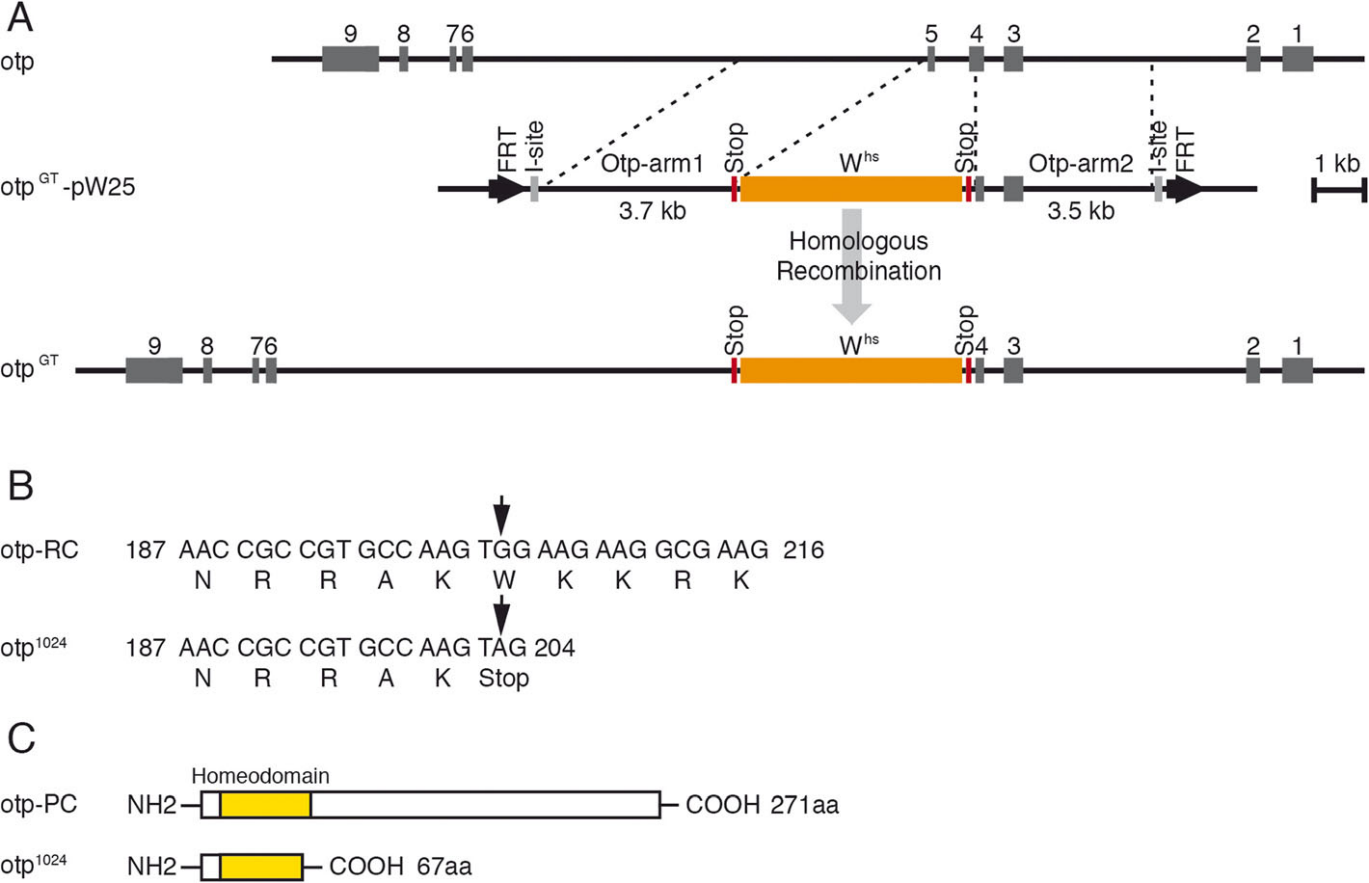
Generation of the *otp* ^GT^ allele and molecular analysis of the *otp*^1024^ allele. **a** At the top, the genomic organization of the *otp* gene is shown with exons indicated as gray boxes. The *otp* gene targeting construct in the targeting vector pW25 (otp^GT^-pW25) is shown below (FRT, FLP recombination target sequences; I-site, I-SceI recognition site; W^hs^, *white* gene with hsp70 promoter). Further down the *otp* genomic region after the gene targeting event is indicated (otp^GT^); here part of exon 4 (containing the translation start) and the complete exon 5 (containing most part of the homeobox) are replaced by the *white* gene of the targeting construct. **b** Nucleotide and amino acid sequences in wild-type (here otp-RC as an example) and mutant DNA. EMS induced a G to A transition in the coding region of *otp*^1024^ leading to the formation of a stop codon. **c** Schematic overview of the wild-type Otp protein with the localisation of the homeodomain (yellow) in comparison to the truncated mutant protein of the *otp*^1024^ allele

So far, no *otp* alleles have been described in previous studies. Thus, in order to analyse the function of *otp* in *Drosophila* development, we generated *otp* alleles by two different approaches. In ends-out gene targeting [40–42], we generated the mutant *otp* allele *otp*^GT^ (Fig. 4a). In this mutant, the translation start site resulting from the translation of the hindgut specific transcript and most of the homeobox of the *otp* gene were missing thereby completely inactivating the *otp* function. The correct gene targeting event was verified by PCR amplification of both ends of the targeting construct.

In a previous EMS-mutagenesis screen [43] for mutants of the 57B region, 33 lethal mutations representing 16 complementation groups were isolated. By complementation analysis using the gene targeting strain *otp*^GT^, we identified two mutant strains (1024 and 13064) not complementing the *otp*^GT^ allele and also each other, suggesting that they represent *otp* alleles. To analyse these alleles at the molecular level, all *otp* exons including the exon-intron boundaries of both strains were PCR amplified and sequenced. Unfortunately, no sequence alterations were identified in strain 13064, but in strain 1024, a single G to A transition in exon 6 induced a change from a tryptophan codon into a stop codon at amino acid position 67 in the carboxy terminus of the homeodomain (Fig. 4b, c). These results verified that the mutant strains 1024 and 13064 represent *otp* alleles, from now on called *otp*^1024^ and *otp*^13064^.

### Lethality and phenotype of *otp* alleles

**Fig. 5:**
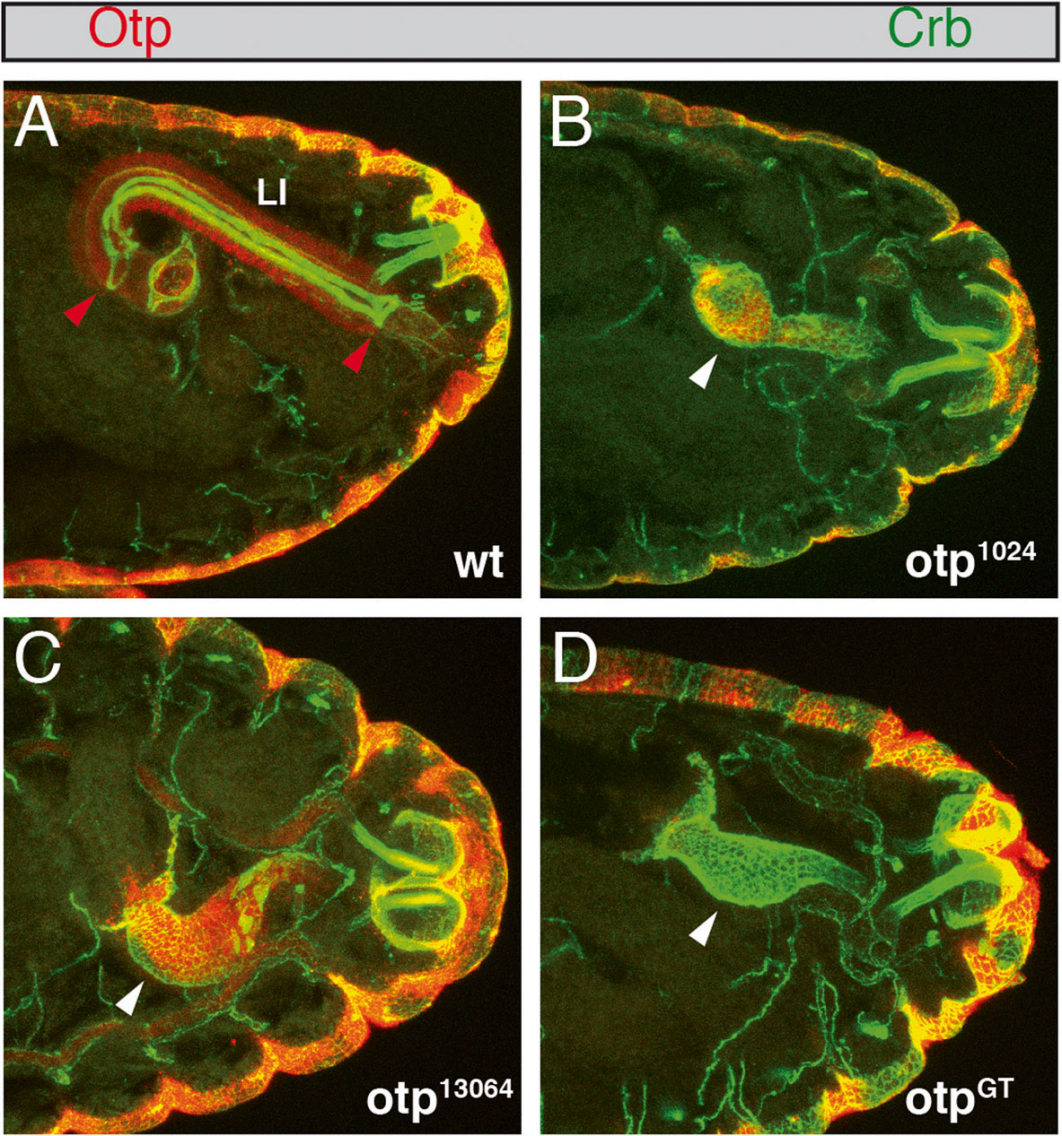
Shortening of the hindgut in *otp* mutants. Anti-Otp- (red) and anti-Crumbs- (Crb) (green) antibody staining of stage 16 wild-type embryos (**a**), homozygous *otp*^1024^ (**b**), *otp*^13064^ (**c**) and *otp*^GT^ (**d**) embryos. In wild-type embryos (**a**) the borders of the large intestine (LI) are marked by red arrowheads, and the shortened hindgut of homozygous *otp*^1024^, *otp*^13064^, and *otp*^GT^ embryos (**b**-**d**) is indicated by white arrowheads. All views are lateral views, anterior is to the left

To determine the strength of each *otp* allele, we analysed their lethality periods by comparing the lethality rates of homozygous *otp*^1024^, *otp*^13064^ and *otp*^GT^ mutant animals with those of wild-type animals. All three *otp* alleles showed embryonic lethality. Furthermore, we investigated the embryonic phenotype that caused that lethality by focusing on the hindgut, the major expression domain of *otp*. We used Crumbs (Crb) which is expressed on the apical surface of ectodermally derived epithelia [44] as a marker in combination with Otp to detect changes in hindgut length. In the hindgut, Crb labels the anterior and posterior boundary cells of the large intestine as well as the borders between the dorsal and ventral domains of the large intestine [8]. In wild-type embryos, the described expression of Crb in the large intestine was clearly visible, and the expression of Otp in the rectum and anal pads in addition to the large intestine was also observed (Fig. 5a). In contrast to wild-type embryos, anti-Otp-antibody staining revealed that homozygous mutant embryos of all three *otp* alleles are characterized by a complete loss of Otp expression (Fig. 5b-d). Crb staining shows changes in hindgut length and morphology in homozygous embryos of all three *otp* alleles (Fig. 5b-d, arrowheads). Compared to the hindgut length of wild-type stage 16 embryos, the hindgut length of homozygous *otp* embryos is only about one third of wild-type length.

**Fig. 6:**
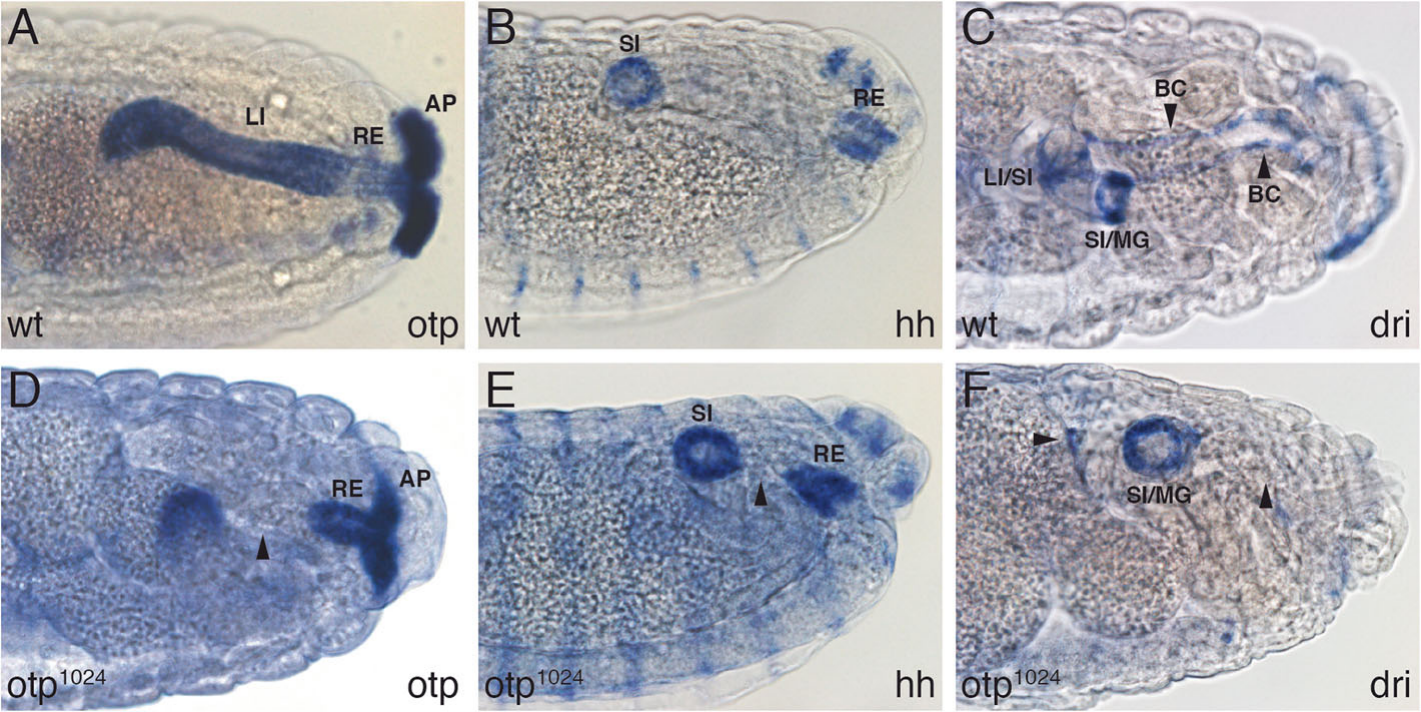
The *otp* hindgut phenotype. Whole mount in situ hybridisation of *otp* (**a**, **d**), *hh* (**b**, **e**) and *dri* (**c**, **f**) mRNA in stage 14 wild-type (**a**-**c**) and *otp*^1024^ mutant embryos (D-F); the probes are indicated on the right, the genotypes of the embryos on the left. In all views, anterior is to the left, all views are lateral views. Compared to wild-type embryos (**a**), the large intestine is almost completely missing in *otp*^1024^ embryos (black arrowhead), and only expression in the rectum and anal pads is visible using an otp probe (**d**). The expression of *hh* in the small intestine and rectum seen in wild-type embryos (**b**) is closer together in *otp*^1024^ mutant embryos (black arrowhead) due to the missing large intestine (**e**). From the expression of *dri* in the border cells and the boundaries between large intestine/small intestine and small intestine/midgut (**c**) only the small intestine/midgut expression is left, the other expression domains are missing (**f**; black arrowheads). AP, anal pads; BC, boundary cells; LI, large intestine; MG, midgut; RE, rectum; SI, small intestine; *dri, dead ringer; hh, hedgehog; otp, orthopedia*

To analyse the effect of a mutation in *otp* more specifically, we used an in situ hybridisation to compare the distribution of the *otp* mRNA from wild-type embryos with *otp*^1024^ mutant embryos. Assuming that the point mutation in *otp*^1024^ embryos has no effect on the mRNA expression, a loss of expression in certain regions might give a first hint for affected areas in *otp* mutants. In stage 14 wild-type embryos otp was expressed in the large intestine, rectum and anal pads (Fig. 6a). In contrast to Otp protein *otp* mRNA could be detected in the remaining part of the hindgut of homozygous *otp*^1024^ embryos (Fig. 6d). Compared to wild-type embryos, the anal pads of *otp*^1024^ embryos were reduced and not located at the posterior end of the embryo. While the rectum was still present, but the large intestine was missing (Fig. 6d, black arrowhead). Additional markers were used to find out which of the different hindgut regions are affected in *otp* mutants*. Hedgehog (hh)* encodes a cell signalling molecule that is required for the maintenance of the small intestine and rectum where it is expressed [8, 9] (Fig. 6b). These two expression domains were not affected in the hindgut of *otp*^1024^ embryos but they are moved further together (Fig. 6e, black arrowhead) indicating that the part between these two domains is affected. The AT-rich interaction domain (AID) box gene *retained (retn) / dead ringer (dri)* was expressed in the boundary cells of the large intestine (Fig. 6c, black arrowheads) and at the border between the large intestine/small intestine and small intestine/midgut [45, 46] (Fig. 6c). No expression of *retn / dri* could be detected in the boundary cells between the ventral and dorsal region of the large intestine and at the posterior border of the large intestine in homozygous *otp*^1024^ embryos (Fig. 6f, black arrowheads) indicating that these cells are missing in *otp* mutants. The other two *otp* alleles also showed a similar hindgut phenotype (data not shown) and therefore can be considered as equally strong. Taken together these results revealed that the small intestine and rectum develop quite normally and showed a normal expression pattern of the markers used in homozygous *otp* mutant embryos, whereas the large intestine was dramatically affected. No expression of large intestine specific genes could be detected, suggesting that this central domain is completely missing resulting in a reduction in hindgut length due to the loss of the large intestine. In addition to the effect in the hindgut, the anal pads were reduced in size and did not reach the posterior end of the embryo.

### Apoptosis in the hindgut primordium of *otp* embryos

**Fig. 7:**
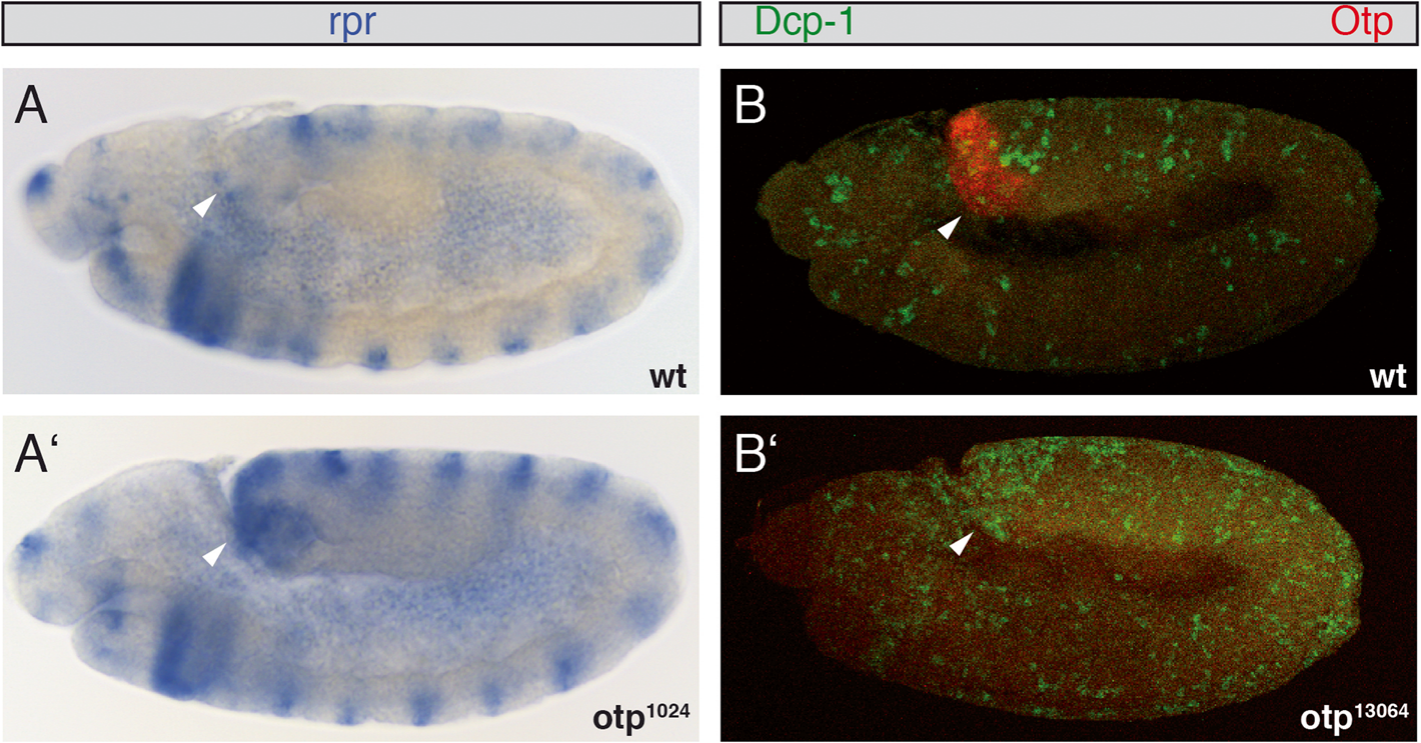
Apoptosis in wild-type and *otp* mutant embryos. Whole mount in situ hybridisation of *reaper* (*rpr*) mRNA in wild-type (**a**) and homozygous *otp*^1024^ mutant embryos (**a’**) at stage 10 (white arrowheads). Antibody staining with anti-Otp antibodies (red) and anti-Cleaved Drosophila Dcp-1 antibodies (green) to highlight apoptotic cells in wild-type (**b**) and *otp*^13064^ mutant embryos (**b’**) at stage 10 (white arrowheads). Stages were determined according to Campos-Ortega and Hartenstein (1997) [1]. All views are lateral views, anterior is to the left

The primary cause of the reduced hindgut and anal pads in *byn* mutants is ectopic cell death in the hindgut primordium [22]. In order to investigate whether the reduced hindgut of *otp* mutants might also be caused by apoptosis, we analysed the expression of the pro-apoptotic gene *reaper (rpr)* in homozygous mutant *otp* embryos (Fig. 7). *Rpr* RNA is expressed in cells about to undergo apoptosis [47]. During hindgut development of wild-type stage 10 embryos, there were some *rpr* expressing cells in the hindgut primordium (Fig. 7a, white arrowhead), but apoptosis does not play a role in determining the size or morphology of the embryonic hindgut in wild-type embryos [5]. At the same stage an increasing number of *rpr* expressing cells were detected in the hindgut primordium of homozygous mutant embryos from all *otp* mutant alleles, here an *otp*^1024^ embryo is shown as an example (Fig. 7A’, white arrowhead). In addition to *rpr* as a cell death marker, we also used an antibody against the *Drosophila* effector caspase Dcp-1 [48] and stained all *otp* mutant strains. Here again an increased amount of Dcp-1 is present in the hindgut primordium of all *otp* alleles compared to the wildtype (Fig. 7b, white arrowhead), and an *otp*^13064^ embryo is shown as an example (Fig. 7B’). These results indicate that the reduced hindgut of *otp* embryos is caused by elevated apoptosis in the hindgut primordium and developing hindgut.

## Discussion

In this paper, we analysed the function of the transcription factor Orthopedia during hindgut development. In the embryo, Otp is expressed in the hindgut primordium, the developing hindgut, and the anal pads. This expression is dependent on several upstream regulators, such as *lines* [5] and *byn* [31]. Byn directly activates *otp* through modular binding sites upstream of hindgut specific promoter of *otp* in a cooperative fashion [31]. Otp is then expressed in the large intestine, rectum and anal pads, unlike Byn, which is also expressed in the small intestine. Byn alone is therefore not sufficient to activate *otp*; *lines* expression might also be needed. In the small intestine where *byn* is expressed, *lines* is repressed by *drumstick* preventing *otp* activation [49]. If *lines* is overexpressed in the small intestine, the repressive effect of *drumstick* can be overruled and *otp* expression can take place (data not shown), which supports the proposed model that *lines* and *byn* are necessary for *otp* expression in the hindgut. Once *otp* is activated in the embryonic hindgut, its expression continues till the adult stage. The only region where *otp* in contrast to *byn* is not expressed is the larval hindgut proliferation zone in the anteriorly located pylorus which supposedly generates hindgut stem cells capable of replacing the larval hindgut cells undergoing apoptosis and building the adult hindgut. The presence of adult hindgut stem cells was questioned by Fox and Spradling (2009) [50], who showed that proliferation only takes place in response to tissue damage. The current view is that all parts of the *Drosophila* intestinal tract maintain stem cells that could migrate across organ boundaries [51]. Since *otp* expression was never shown in areas where intestinal stem cells are present, *otp* is rather expressed in and a marker for differentiated hindgut cells.

To analyse the function of *otp*, we generated a mutant allele by gene targeting via homologous recombination and using this targeting strain, we identified two additional mutant alleles by complementation among our EMS-induced collection of mutants from the 57B region. In the gene targeting mutant, part of exon 4 and exon 5 were missing resulting in an N-terminal deletion of the otp-PC protein form including most of the homeodomain. In the *otp*^1024^ mutant, the N-terminus and most of the homeodomain were present, but the C-terminal part of the protein was missing. In both cases no Otp protein expression was detected since our anti-Otp antibody was directed against the C-terminal part of Otp. In the case of *otp*^13064^, we could also not detect a protein with our anti-Otp antibody nor detect a sequence alteration in the coding region. The splice sites were intact, but we cannot rule out the possibility that a cryptic splice site might be generated. The generation of cryptic splice sites by mutations is often the case in human genetic disorders like Neurofibromatosis type I (NFI) [52] or Cystic fibrosis (CF) [53] where such mutations generate pseudo-exons (see [54] for review). Another possibility might be a mutation in a regulatory region of the gene. All *otp* alleles showed embryonic lethality with a strong hindgut phenotype. The loss of the large intestine led to a dramatic reduction in hindgut length to about one third of the wildtype length. This phenotype is comparable to the *byn* phenotype, since *byn* is directly regulating *otp* [31]. The three transcriptional regulators *drm*, *bowl* and *lin* are required for patterning and cell rearrangements during elongation in the hindgut, but when compared to *otp*, showed only a reduction to 40% (*drm* and *bowl*) or 50% (*lin*) in the mutant phenotype [5], suggesting that the loss of *otp* function is much more severe compared to these genes. A gut specific function of *otp* like seen in *Drosophila* is not known for *otp* genes in higher organisms, where an expression in the nervous system and a function in various aspects of nervous system development is known. In *Drosophila*, *otp* is also expressed in the ventral nerve cord and the brain. Expression in the nerve cord seems to be post-transcriptionally regulated, since, in contrast to the mRNA expression posterior to segment A2, the Otp protein is not expressed there. This might be due to the regulation via a miRNA as it was seen for other developmental processes (see [55] for review).

The nervous system function of *otp* in higher organisms has been mainly analysed in various model organisms like zebrafish and mice. Here it was shown that Otp is expressed in the hypothalamus that exerts influence on physiological function in various processes like blood pressure, circadian rhythmicity, energy balance and homeostasis [37]. In zebrafish Otp in the hypothalamus is required in the preoptic area for the production of the neurohypophysial peptide arginine vasotocin [56], for dopaminergic and neuroendocrine cell specification [57, 58], regulation of stress response [59, 60] and through neuropeptide switching that impacts social behavior [61]. In mice, a loss of Otp leads to a progressive impairment of neuroendocrine cells in the hypothalamus, and homozygous mutant animals die soon after birth with a failure of terminal differentiation of neurosecretory cells [62, 63]. Recently, it was shown that a mutation in Otp is associated with obesity and anxiety in mice [64]. The *OTP* gene from humans was cloned some time ago [36], but only during the last few years, it could be shown that OTP has a high diagnostic value concerning pulmonary neuroendocrine tumours. Even if these tumours accounted for only 1–2% of all lung tumours, their occurrence increased over the last decades [65]. People with poor survival rates showed a strong downregulation of *OTP* [66]. OTP that is normally located in the nucleus (nOTP) could also be detected in the cytoplasm (cOTP) or not be present at all. Patients with nOTP have a favourable disease outcome, those with cOTP have an intermediate outcome and those with no OTP expression have the worst disease outcome [67] demonstrating the diagnostic value of OTP. Due to these very interesting aspects of Otp function in the nervous system of higher organisms, it would be interesting to analyse the function of Otp during embryonic brain development of *Drosophila*, as well as later functions of Otp during larval development and in the adult, using our newly generated *otp* alleles in the future.

## Conclusions

Using gene expression analysis and newly generated mutant *otp* alleles, we showed that the *Drosophila* homeodomain transcription factor Orthopedia is an important factor for hindgut development. Our findings demonstrate a requirement of *otp* to build the large intestine of the hindgut and also in the correct formation of the anal pads. This phenotype is caused by apoptosis at the beginning of hindgut development. Otp as a downstream factor of *byn* is most likely present only in differentiated hindgut cells during all stages of development rather than in stem cells.

## Methods

### Whole-mount in situ hybridisation

To synthesise DIG-labeled DNA probes the following DNAs were used: A *dri/rtn* cDNA clone (LD35748, BDGP), a *hh* cDNA clone [68], an *otp* cDNA clone containing exons 3–9 (otp-RC), a 1.6 kb genomic *otp* clone containing exons 1 and 2, an *otp* fragment containing exon 3 amplified with the primers 10,036–1 (5′-TCGCACCTCGGTTTCTTCTAC-3′) and 10,036–2 (5′-GGCAATAGTTATTCCACCGATC-3′), a 0,8 kb fragment of the *reaper* gene amplified with the primers rpr1 (5′-GTCATTGAATAAGAGAGACACC-3′) and rpr2 (5′-GCAATTTTTAGCCAACTTCGAC-3′) and a 1.0 kb fragment of the *byn* gene amplified with the primers byn1 (5′-GTTCCTGGTGGCAAGGCAGAG-3′) and byn2 (5′-AGATGTTGCGGCCACTGCACAC-3′). In situ hybridisation to whole-mount embryos was performed according to Tautz and Pfeifle [69] with slight modifications [70].

### *Drosophila melanogaster* stocks

The following stocks were used: *byn*^apro^ (enhancer-trap line) [10] and *yw*^67c23^. The latter was used as the wild-type stock in this study. Stocks were raised on standard medium at 25 °C.

### Antibody production

To generate anti-Otp antibodies against the Otp C-terminus a 0.6 kb fragment of our *otp* cDNA cW26/1 (corresponding to otp-RC) was amplified using the primer 10036–13 (5′-GGATCCACCAATGTCTTCCGCACCC-3′) to add a *Bam*HI site (underlined) and the primer 10036–14 (5′-GAATTCGAATTGTAGTGTTCGTAGTTGTGTGG-3′) with an *otp Eco*RI site (underlined). The fragment was subcloned into the vector pCRII-TOPO (Invitrogen, Carlsbad, California, USA), cut with *Bam*HI and *Eco*RI as a 577 bp fragment (position 886–1463 of CG10036-RC, Flybase) and cloned in frame into the pGEX-4 T1 expression vector (Amersham, Buckinghamshire, United Kingdom). The fusion protein of glutathione-S-transferase and Otp was purified as described [71]. Immunisation of guinea pigs was done by Pineda Antibody Service (Berlin, Germany).

### Immunostaining

Embryos were collected, dechorionated with 50% bleach for 2 min, washed with 0.1% NaCl / 0.1% Triton X-100 and fixed for 12 min in 3.7% formaldehyde in PEM (100 mM PIPES, 1 mM EGTA, 1 mM MgCl_2_) and heptane. After removal of both phases, embryos were devitelinised in equal volumes of heptane and methanol by 2 min of vigorous shaking and washed three times with methanol. The 3rd instar larvae and adult hindguts were dissected in 1x phosphate buffered saline (PBS), fixed for 60 min in 2% paraformaldehyde in PBL and washed three times with 1x PBS containing 0.2% Triton X-100 (PBX) and then incubated for 3 × 5 min in methanol. Fixed embryos or larvae were washed 3 × 30 min and 6 × 30 min in PBX and blocked for 30 min in 5% normal horse serum and 10% PBX in PBS. Incubations with primary antibodies were done overnight at 4 °C. Samples were washed 3x5min and 6 × 30 min in PBX and blocked for 30 min in 5% normal horse serum and 10% PBX in PBS. After an overnight incubation with secondary antibodies at 4 °C embryos or larvae were washed 3 × 30 min and 6 x 30 min in PBX and mounted in Vectashield (Vector Laboratories). Images were obtained using an Olympus BX61 microscope (Olympus, Hamburg, Germany) for bright field and DIC microscopy or a Leica TCS SP5 microscope (Leica, Wetzlar, Germany) and a ZEISS LSM 710 microscope (Carl Zeiss AG, Oberkochen, Germany) for laser confocal microscopy. Images were processed using FIJI and ImageJ (NIH. Md., USA), Adobe Photoshop and Adobe Illustrator (Adobe Systems, San Jose, CA, USA).

The primary antibodies used were: rabbit anti-β-Galactosidase antibody (1:1000, Cappel), mouse anti-Crumbs antibody Cq4 (1:5, [44], DSHB), rabbit anti-Cleaved Drosophila Dcp-1 antibody (1:100, Cell Signaling), guinea pig anti-Orthopedia (1:400, this work). Secondary antibodies were goat anti-mouse, anti-rabbit and anti-guinea pig, conjugated with Alexa 488 or 568 (1:1000, Molecular Probes, Eugene, Oregon, USA).

### Generation of an *otp* mutant by ends-out homologous recombination

The allele *otp*^GT^ was generated as previously described [42]. The primers OtpGT3 (5′-GGCGCGCCTCAAAATAGGGCTTAAAACA-3′) and otpGT4 (5′-GGCGCGCCATAAACAGAATGCGTGCCAG-3′) were used in a PCR with BACR10P11 DNA as the template to amplify a 3.5 kb fragment (Otp-I) with *Asc*I sites on both ends (underlined). The fragment was cut with *Asc*I and cloned into the *Asc*I site of the pW25 targeting vector (DGRC) to generate pW25/OtpI. Next, primers OtpGT1 (5′-GCGGCCGCATCGTAGTTTGCCAACTCGAGG-3′) and OtpGT2 (5′-GGTACCGCCGGATATGTGCAGGCGTG-3′) were used in another PCR with BACR10P11 DNA to add *Not*I and *Acc*65I sites (underlined) to a 3.7 kb fragment (OtpII), which was then cutted with *Not*I and *Acc*65I and cloned into the corresponding sites of the vector pW25/OtpI to generate pW25/OtpI+II. Four independent lines were obtained by germline transformation with the pW25/OtpI+II donor construct using standard techniques [72]. A donor target strain with the construct on the X-chromosome was crossed to *yw; 70FLP, 70I-SceI, Sco/CyO* flies and the F1 progenies were heat-shocked at 37 °C for 1 h on days 3, 4 and 5 after laying eggs. Among 106 crosses we identified red eyed flies in two crosses. One fly line, *otp*^GT^ was balanced and the correct gene targeting event was verified by PCR amplification with genomic DNA extracted from *otp*^GT^ flies as template and primers P1 (otp; 5′-CGTCCGGCACTTTGGCACG-3′) and P2 (pW25 vector; 5′-CGTGCTCATCGCGAGTACG-3′) or P3 (pW25 vector; 5′-GAGTGCCGTTTACTGTGCG-3′) and P4 (otp; 5′-GAGCAGCCCAGATTCCATGC-3′).

### Amplification and cloning of the coding region of *otp* alleles

Amplifications of the coding region including the splice sites were performed using genomic DNA from fly stocks *otp*^1024^ and *otp*^13064^. For polymerase chain reactions Taq Polymerase from ThermoFisher Scientific (Waltham, Massachusetts, USA) was used according to supplier’s instructions. PCR products were sequenced by Starseq (Mainz, Germany). The region showing a sequence alteration in *otp*^1024^ DNA compared with the wild-type sequence was again PCR-amplified using more closely located primers. For *otp*^1024^ DNA the primers 1024–5 (5′-CTACCTTTAAATCTGTGGGTTTTGATGC-3′) and 1024–6 (5′-CATGCCAGGCGAAAAAGTGTC-3′) were used. The PCR product was subcloned into the TOPO vector pCR2.1 (ThermoFisher Scientific, Waltham, Massachusetts, USA), and at least 10 individual clones from PCR product cloning were checked by sequencing. Since the *otp*^1024^ DNA was generated from heterozygous flies approximately 50% of the clones showed the wild-type sequence and 50% the altered sequence due to the point mutation.

## Data Availability

The datasets supporting the conclusions of this article are included within the article. Materials are available from the corresponding author on reasonable request.

## Abbreviations

BDPD: Berkeley Drosophila Genome Project
cDNA: complementary desoxyribonucleic acid
DSHB: Developmental Studies Hybridoma Bank
EST: expressed sequence tag
